# Tick-borne encephalitis vaccine effectiveness and barriers to vaccination in Germany

**DOI:** 10.1038/s41598-022-15447-5

**Published:** 2022-07-09

**Authors:** Teresa M. Nygren, Antonia Pilic, Merle M. Böhmer, Christiane Wagner-Wiening, Ole Wichmann, Thomas Harder, Wiebke Hellenbrand

**Affiliations:** 1 Immunisation Unit, Robert Koch Institute, Seestraße 10, 13353 Berlin, Germany; 2 Charité - Universitätsmedizin Berlin, Berlin, Germany; 3 Bavarian Health and Food Safety Authority (LGL), Oberschleißheim, Germany; 4 Institute of Social Medicine and Health Systems Research, Otto-Von-Guericke-University Magdeburg, Magdeburg, Germany; 5 State Health Office Baden-Wuerttemberg (LGA), Stuttgart, Germany

**Keywords:** Viral infection, Central nervous system infections, Vaccines

## Abstract

Tick-borne encephalitis (TBE) vaccination coverage remains low in Germany. Our case–control study (2018–2020) aimed to examine reasons for low vaccine uptake, vaccine effectiveness (VE), and vaccine breakthrough infections (VBIs). Telephone interviews (581 cases, 975 matched controls) covered vaccinations, vaccination barriers, and confounders identified with directed acyclic graphs. Multivariable logistic regression determined VE as 1—odds ratio with 95% confidence intervals (CI). We additionally calculated VE with the Screening method using routine surveillance and vaccination coverage data. Main vaccination barriers were poor risk perception and fear of adverse events. VE was 96.6% (95% CI 93.7–98.2) for ≥ 3 doses and manufacturer-recommended dosing intervals. Without boosters, VE after ≥ 3 doses at ≤ 10 years was 91.2% (95% CI 82.7–95.6). VE was similar for homologous/heterologous vaccination. Utilising routine surveillance data, VE was comparable (≥ 3 doses: 92.8%). VBIs (n = 17, 2.9% of cases) were older, had more comorbidities and higher severity than unvaccinated cases. However, only few VBIs were diagnostically confirmed; 57% of re-tested vaccinated cases (≥ 1 dose, n = 54) proved false positive. To increase TBE vaccine uptake, communication efforts should address complacency and increase confidence in the vaccines’ safety. The observed duration of high VE may inform decision-makers to consider extending booster intervals to 10 years.

## Introduction

From 2017 to 2020, 529 annual cases of tick-borne encephalitis (TBE) were notified in Germany^1^. In Germany, TBE vaccination is recommended to people with tick exposure living in, traveling to, or working in risk areas.

TBE virus infections have been vaccine-preventable since the 1970s, with two vaccines currently licensed in Germany (ENCEPUR (Bavarian Nordic) and FSME-IMMUN (Pfizer)^2^. However, complete (≥ 3 doses) and on-time vaccination coverage is only 22.3% in Bavaria and 18.0% in Baden–Wuerttemberg^1^. These southernmost federal states give rise to ~ 85% of TBE cases in Germany^1^. This project addresses key aspects of TBE vaccination: vaccination barriers, vaccine effectiveness (VE), and vaccination breakthrough infections (VBI).

First, vaccination barriers like poor risk perception are known from Finland^3^ and Sweden^4^, but have to our knowledge never been studied in larger samples in Germany. Currently, 175 TBE risk districts exist in Germany and 71% of cases occur within 58 areas with highest incidence^1^. Coverage (≥ 3 doses, last dose on-time) is 22.3% in these areas, only somewhat higher than in 58 low-incidence areas at 17.6%^1^. Identifying key barriers is crucial for designing public health campaigns to increase coverage, particularly in high-incidence areas.

Second, VE has hitherto mainly been determined in Austria^5^. Results may however not be transferrable to other European countries given Austria’s uniquely high vaccination coverage at 85% for ≥ 1 dose^6^. Coverage (≥ 1 dose) in 10 other TBE-endemic European countries averages 25% according to a 2015 cross-sectional online survey (Germany: 27%)^6^. VE calculated at high coverages of > 80% like in Austria is prone to confounding, as unvaccinated persons tend to differ from the source population in covariates linked to disease risk that are independent of vaccination^7^. This applies to case–control-designs^7^ and likely also other designs, as the unvaccinated population fraction becomes ever more selected at higher coverages and may increasingly differ from the vaccinated in potentially confounding parameters. Recently, VE was estimated in Latvia and Southern Germany^8^, but data sources had weaknesses. Vaccination data lacked details on vaccine type and dose timing (except last dose) and coverage data originated from the above-mentioned cross-sectional survey^6^ whose online design is prone to selection bias. An additional methodological shortcoming of both previous studies^5,8^ is using the basic formula of the Farrington Screening method^9^, as provided in the “Methods” section below, which does not permit confounder adjustment beyond stratification into sub-groups. We therefore aimed to determine vaccine-/dose-/timing-specific VE based on a carefully adjusted case–control study to provide more robust VE estimates.

Thirdly, we aim to explore acute TBE severity in VBI cases. There is uncertainty whether severity differs from that in unvaccinated cases^10–15^. Moreover, we examined severity in patients with incomplete vaccination series (1–2 doses) to rule out that this constellation leads to enhanced disease^16^. Antibody-dependent enhancement (ADE) has been discussed, but appears overall unlikely in TBE^17^, yet research on humans is sparse (e.g. Ref.^10^). Notably, serum diagnostics are challenging in previously TBE vaccinated TBE cases due to unspecific antibody rises and cross-reactivity^18^. German routine surveillance therefore stipulates validating vaccinated cases (≥ 1 dose) at the national reference laboratory with the NS1-antibody test^19^ to distinguish between true VBIs and false positives. We report validation results within our sample.

In summary, detailed insights on vaccination barriers, VE, and VBIs are valuable for public health planning, practicing physicians, and campaigns aiming to diminish the substantial morbidity still caused by this preventable infection.

## Results

### Participant characteristics

In total 581 of 1,220 eligible cases (48%) participated, without indication of selection bias (see Ref.^20^). Matching factors were similar between cases and controls; vaccination status differed markedly (Table 1).

**Table 1:** Participant characteristics: demographics, TBE vaccination status, and covariates required to adjust vaccine effectiveness analysis (see Supplementary Fig. 2).

Demographics	Cases	Controls	p-value
	*n* = 581^a^	*n* = 975	
	n (%)	n (%)	
Male	368 (63.3%)	608 (62.4%)	0.699
Age group 2–13 years	53 (9.1%)	60 (6.2%)	0.091
Age group 14–65 years	407 (70.1%)	702 (72.0%)	
Age group ≥ 65 years	121 (20.8%)	213 (21.9%)	
≥ 1 comorbidity (self-reported)	118 (21.2%)	236 (24.2%)	0.172
**Highest level of completed secondary education**^**b**^** (duration in years)**			
Abitur (12–13 years)	162 (29.0%)	307 (31.5%)	0.049
Fachabitur (12–13 years)	55 (9.9%)	81 (8.3%)	
Realschulabschluss (10 years)	142 (25.4%)	300 (30.8%)	
Hauptschulabschluss (9 years)	133 (23.8%)	194 (19.9%)	
Still in school/none/missing	66 (11.8%)	93 (9.5%)	
**TBE-vaccination**			
Unvaccinated	497 (85.5%)	397 (40.7%)	< 0.001
Any TBE vaccination (≥ 1 dose)	78 (13.4%)	578 (59.3%)	
Unvaccinated, but received first dose after symptom onset	6 (1.0%)	–	
Among TBE vaccinated: vaccination card in interview	59 (79.7%)	415 (71.8%)	0.149
**TBE-vaccination: interval since last dose**			
Unvaccinated	497 (86.4%)	397 (40.7%)	< 0.001
≥ 3 doses, on-time	17 (3.0%)	235 (24.1%)	
≥ 3 doses, not on-time, ≤ 10 years	12 (2.1%)	106 (10.9%)	
≥ 3 doses, not on-time, > 10 years	7 (1.2%)	43 (4.4%)	
2 doses, on-time	4 (0.7%)	8 (0.8%)	
1–2 doses	33 (5.7%)	116 (11.9%)	
≥ 1 dose, additional data missing	5 (0.9%)	70 (7.2%)	
**TBE-vaccination: vaccine type**			
≥ 3 doses ENCEPUR	8 (1.4%)	105 (10.8%)	0.411
≥ 3 doses FSME-IMMUN	13 (2.3%)	106 (10.9%)	
≥ 3 doses, heterologous schedule	7 (1.2%)	101 (10.4%)	
**TBE-vaccination: timing of primary immunisation**			
≥ 3doses, standard timing	11 (1.9%)	132 (13.5%)	0.671
≥ 3doses, irregular timing	12 (2.1%)	173 (17.7%)	
**Covariates required to adjust VE analysis (see Supplementary Fig.**2**)**			
Rural residence (< 5000 inhabitants)	268 (48.0%)	413 (42.4%)	0.095
Tick bites: never	103 (18.5%)	300 (30.8%)	< 0.001
Tick bites: last bite > 1 year ago	87 (15.6%)	407 (41.7%)	
Tick bites: 1–2 bites in last year	198 (35.5%)	181 (18.6%)	
Tick bites: ≥ 3 bites in last year	170 (30.5%)	87 (8.9%)	
Gardening ≥ 4 × /week^c^	157 (28.1%)	125 (12.8%)	< 0.001
Taking walks ≥ 4 × /week^c^	328 (58.8%)	458 (47.0%)	< 0.001
Other outdoor activity ≥ 4 × /week^c^	179 (32.1%)	253 (25.9%)	0.010
Not staying on paths^c^	133 (23.8%)	100 (10.3%)	< 0.001

^a^558 cases and all controls had interview data, used as denominator for solely interview-derived variables (education and parameters from rural residence onwards).

^b^English translations: Abitur = general qualification for university entrance; Fachabitur = subject-related entrance qualification; Realschulabschluss = intermediate school-leaving certificate; Hauptschulabschluss = completion of compulsory basic secondary schooling.

^c^Cases: within 4 weeks before onset, controls: during reference time. Analysis used 3 levels for frequency-graded covariates: < 1×/week, 1–3×/week, ≥ 4×/week.

Routine surveillance data on vaccination status was available for 566 cases (97.4%). Study data showed excellent agreement with routine data for 99.8% of unvaccinated cases. Yet, 24 of 76 vaccinated cases (31.6%) were misclassified as unvaccinated in routine data.

### Vaccination barriers

**Figure 1:**
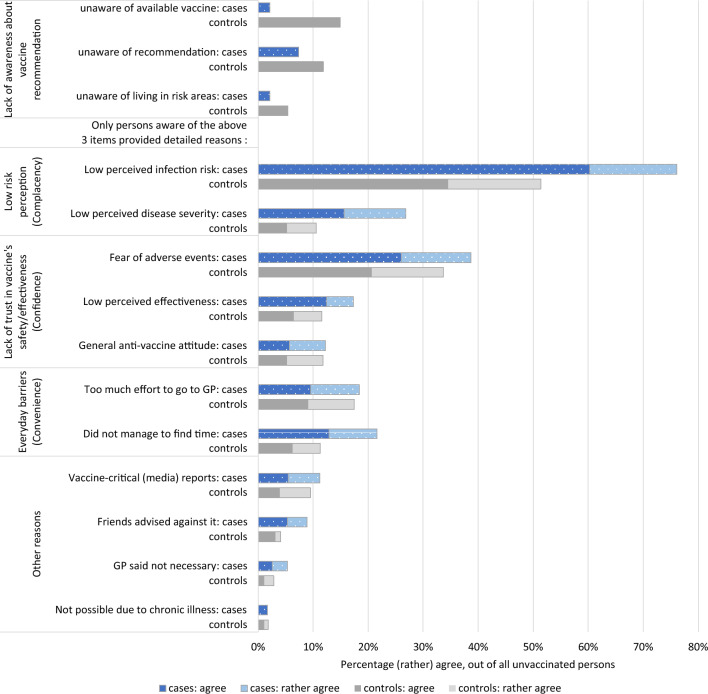
Vaccination barriers reported by unvaccinated cases (*n* = 473) and controls (*n* = 389) living in or visiting TBE risk areas. Multiple answers were possible, except on the first 3 items.

Vaccination barriers were similar in cases and controls, mainly relating to low-risk perception regarding the disease and fear of adverse events following immunization (Fig. 1). Additional reasons were reported by 46 controls and 59 cases, most frequently: never having had tick bites (*n* = 31) or considering the vaccination unnecessary (*n* = 22). Worry about costs or vaccine unavailability were not named.

As the proportion of unvaccinated differed markedly (cases: 85.5%, controls: 40.4%), we compared further characteristics to assess group comparability. Most covariates were similar, while small differences were observed for age and education (Supplementary Table 2).

### VE based on the case–control approach

**Figure 2:**
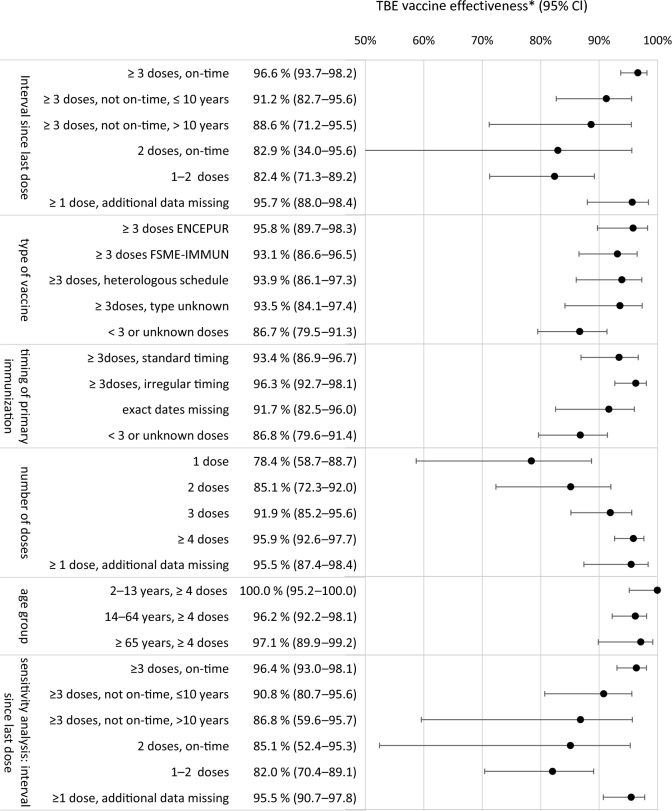
TBE vaccine effectiveness determined by time intervals since last dose, type of vaccine, timing of the first 3 doses, age group, and number of doses, (*n* = 570 TBE cases, 964 controls). The lower section shows a sensitivity analysis of the time interval analysis only using exact dates (no imputation, see “Methods”). Estimates represent the adjusted total causal effect for each TBE vaccination covariate on the outcome TBE. The minimal adjustment set consisted of: matching factors (age, sex, region), dog ownership, tick bites, risk behaviours (taking walks, gardening, other outdoor activities, not staying on paths) during 4-week periods of exposure time (cases) or reference time (controls), season, and rural residence (Supplementary Fig. 2). For univariable estimates and case numbers in each category, see Supplementary Table 1. *CI* confidence interval, *VE* vaccine effectiveness.

VE after ≥ 3 doses with the last dose on-time was 96.6% (Fig. 2). When time intervals were exceeded (> 3 or > 5 years), but the last dose was within 10 years, VE was 91.2%. For “ ≥ 3 doses, last dose 5–10 years ago” (11 cases, 56 controls), VE was 82.4% (95% CI 63.0–91.7%). If the last dose was > 10 years ago, VE was 88.6%. A sensitivity analysis only using exact vaccination dates produced almost identical results (Fig. 2). For “≥ 3 doses, on-time” and “≥ 3 doses, not on-time, ≤ 10 years” combined, VE was 95.2% (95% CI 92.2–97.0) and remained stable across age groups (99.8%, 95.7%, and 95.3% for  < 18, 18–64 and ≥ 65 years, respectively).

VE at ≥ 3 doses was similar for homologous vaccination series with either ENCEPUR (95.8%) or FSME-IMMUN (93.1%) and heterologous vaccination (93.9%) (Fig. 2). VE was not reduced when primary immunisation had irregular timing. VE increased gradually with dose number and ≥ 4-dose-VE was comparable across age groups (Fig. 2).

### VE based on the screening method

VE for ≥ 3 doses on-time was 92.8% and in the sensitivity analysis 89.4% (Table 2).

**Table 2:** Vaccine effectiveness determined with Farrington’s screening method^9^ and input data for cases (source: routine TBE surveillance 2012–2020) and vaccination coverage 2019 (source: claims data^21^).

Scenario	Unvaccinated cases	Fully vaccinated cases	Population proportion unvaccinated (%)	Population proportion fully vaccinated (%)	Vaccine effectiveness
Raw routine data	2,529	56	62.04	18.98	92.76
Corrected for misclassification	2,529	82	62.04	18.98	89.42

### Vaccination breakthrough infections

There were 17 VBIs (2.9% of 581 cases): ten had received 3–4 doses, four 5–6 doses, and three 8–9 doses. Ten VBIs had homologous vaccination series (6 FSME-IMMUN, 4 ENCEPUR), four had heterologous series, three lacked data. The median interval between last dose and onset was 1.8 years (range 70 days–4.1 years). VBIs were older with greater comorbidity than unvaccinated cases (Table 3). Illness- or medication-induced immunosuppression could not explain VBIs. TBE was more severe in VBIs than in unvaccinated cases (Table 3). VBIs did not cluster spatially (Supplementary Fig. 3) or temporally.

**Table 3:** Characteristics and acute TBE manifestations in previously unvaccinated TBE cases, cases with vaccination breakthrough infections, and cases with incomplete TBE vaccination at 1–2 doses.

Age group	Unvaccinated cases	VBI cases	Cases with 1–2 doses	p-value	p-value
	*n* = 497^a^	*n* = 17^a^	*n* = 37^a^	VBI vs. unvaccinated	1–2 doses vs. unvaccinated
	n (%)	n (%)	n (%)		
2–13 years	48 (9.7%)	1 (5.9%)	3 (8.1%)	**0.037**	0.058
14–64 years	345 (69.4%)	8 (47.1%)	32 (86.5%)		
≥ 65 years	104 (20.9%)	8 (47.1%)	2 (5.4%)		
**Demographics**					
Male	314 (63.2%)	9 (52.9%)	23 (62.2%)	0.390	0.902
≥ 1 comorbidity^b^	193 (38.8%)	12 (70.6%)	17 (45.9%)	**0.009**	0.393
Immunosuppression in exposure time	13 (2.6%)	1 (5.9%)	0 (0.0%)	0.128	0.500
**Acute TBE severity**^c^					
Mild	102 (20.5%)	0 (0.0%)	9 (24.3%)	**< 0.001**	0.255
Moderate	306 (61.6%)	6 (35.3%)	18 (48.6%)		
Severe	89 (17.9%)	11 (64.7%)	10 (27.0%)		
**Clinical characteristics**					
Biphasic course	197 (39.6%)	10 (58.8%)	21 (56.8%)	**< 0.001**	**< 0.001**
Hospitalised	443 (89.1%)	17 (100.0%)	33 (89.2%)	0.151	0.992
Median hospital stay (days, range)	10 (1–84)	14 (6–90)	10.5 (2–40)	–	–
ICU admission	54 (10.9%)	8 (47.1%)	6 (16.2%)	**< 0.001**	0.320
**RANKIN score**^22^** and interval between symptom onset and measuring RANKIN**					
Median interval (days, interquartile range)	93 (66–144)	96 (66–198)	100 (76–177)	–	–
0: No symptoms	243 (50.8%)	3 (20.0%)	15 (42.9%)	**< 0.001**	0.629
1: No significant disability	145 (30.3%)	1 (6.7%)	13 (37.1%)		
≥ 2: Slight disability, or worse	90 (18.8%)	10 (66.7%)	7 (20.0%)		

Significant values are in bold.

^a^Of these, 478 unvaccinated cases, 15 VBI cases, and 35 cases with 1–2 doses had interview data. These denominators were used to calculate proportions within purely interview-derived variables (RANKIN score).

^b^From medical data sources and self-reported, for details see Ref.^20^.

^c^As defined in Ref.^20^.

Severity at 1–2 doses was comparable to unvaccinated cases (Table 3). Of 34 cases with reported time interval between last dose and onset, the median interval was 4.5 years (range 1 day–39.6 years). Severity in seven cases with intervals < 30 days was similar to unvaccinated cases (1 mild, 5 moderate, 1 severe).

### Diagnostic validation of vaccinated cases

Validation was only performed on 54 of 108 vaccinated cases (≥ 1 dose). The remaining 54 could not be re-tested, as no samples were sent to the laboratory. Of the 54 re-tested cases, 23 (42.6%) were confirmed and 31 (57.4%) proved false positive. The latter were not eligible to participate. Only 4 of 17 VBIs were confirmed; the remaining 13 could not be re-tested.

## Discussion

This large case–control study enables novel insights on key TBE vaccination aspects: vaccination barriers, VE, and VBI. The high degree of case–control similarity indicates successful matching on potential confounders. The proportion of fully vaccinated controls at 24.1% only slightly exceeds vaccination coverage in Baden-Wuerttemberg (18.0%) and Bavaria (22.3%)^1^, suggesting low risk of pronounced selection bias for TBE-vaccinated, particularly health-conscious controls.

### Vaccination barriers

Cases and controls were similar regarding vaccination barriers, but also other characteristics, lending strength to these results. Small-scale group differences should not be overinterpreted, as recall bias may apply, given the retrospective design. The main barrier, low risk perception, was reported by 50–75%, congruent with Finnish^3^ and Swedish reports^4^. It is true that TBE incidence is low with 0.9–1.9 notifications per 100,000 inhabitants in Southern Germany in 2021^1^. While some risk factors for severe TBE are known^20^, prediction of severity is, however, not possible at the individual level. Risk communication could therefore emphasise the 4–9 × higher number of unreported (subclinical) TBE infections^23,24^ and the limited possibility of predicting who will experience severe disease. Recent research moreover revealed a higher than previously assumed proportion of symptomatic TBE cases with moderate/severe illness, even among children^20^.

Fear of adverse events following immunisation was the second key barrier (~ 35%). Given the excellent safety of both TBE vaccines licensed in Germany^25^, this finding exposes misinformation that could be rectified by information campaigns. In keeping with reimbursement of TBE vaccination in Germany, cost was not a concern, contrasting a Swedish study, where this worry was similarly frequent as low risk perception^4^. The most effective way to increase the low TBE vaccination coverage in Germany would be a large-scale vaccination programme similar to Austria’s, which led to vaccination coverages above 80%. Until such a programme exists, public health efforts aiming to increase vaccine uptake should prioritize informing about TBE risk, potential severity, and vaccine safety and effectiveness, especially in high-incidence areas.

### Vaccine effectiveness

VE for ≥ 3 doses with the last dose on-time was 96.6%. When using the Screening method, VE was slightly lower (89.4–92.8%). The similarity with both methods indicates that confounding may not be a major threat to validity in TBE VE. Thus, the Screening method is suitable for monitoring TBE VE. Our VE results compare well to previous estimations: 99% VE for ≥ 3 doses in Austria^5^, 95.4% ≥ 4 doses in Southern Germany^8^ and 98.9% for ≥ 3 doses in Latvia^8^.

Dose schedule adherence is low in Germany^26^, reflected here by 15.3% of controls with ≥ 3 doses but missed boosters, compared to 24.1% of controls with ≥ 3 doses plus boosters. VE at “≥ 3 doses, not on-time, last dose ≤ 10 years ago” remained high at 91.2%. When the last dose was > 5–10 years ago, VE was still 82.4%. The overall ≥ 3 dose-VE including both on-time and last dose ≤ 10 years was 95.2% and stable across age groups. VE dropped to 88.6% for last dose > 10 years. Finding that VE at ≥ 3 doses persists for ≤ 10 years or longer agrees with seropersistence studies, finding mostly high seropositivity rates at ≥ 4 doses after 10 years^27,28^. Lasting immunity may also relate to rapid secondary antibody response^27^. Our results support the extension of booster intervals to 10 years also in Germany, as under discussion elsewhere^27^ and already implemented in Switzerland and Finland, without observed increases in VBIs^29^.

VE for two on-time doses (up to 1 year, Refs.^30,31^) at 82.9% was lower than previously reported at 97.2–98.7%^5,8,32^. This result is limited by low statistical power: we included four “2-doses-on-time” cases; other studies similarly contained 2 or 11 such cases^8,32^. Other explanations for our lower estimate could be lacking confounder adjustment in previous studies or varying proportions of false positive vaccinated cases.

VE at ≥ 3 doses was similar for homologous vaccination or heterologous vaccination. This reinforces that vaccines can be used interchangeably if necessary^33^. Irregularly timed primary immunisation did not adversely affect VE. Irregular timing was common, and underlines the relevance of population-based VE research with imperfect real-life conditions, allowing transfer of insights to practice.

### Vaccine breakthrough infections

As VBIs (2.9% of cases) did not cluster spatially, VBIs in Germany are unlikely to be caused by local virus variants that escape vaccine-induced immunity. Acute severity in incompletely vaccinated cases was the same as in unvaccinated cases, even at < 30 days since the last dose, providing no indications of ADE^17^. Finding higher severity and symptom persistence in VBI cases than unvaccinated cases may partly be explained by VBIs’ higher age and comorbidity prevalence, which are known severity predictors^20^. Importantly, diagnostics in TBE-vaccinated cases are often unreliable^18^, as 57% of re-tested cases^19^ proved false positive. As only 4 of our 17 VBIs were validated, several of the remaining 13 cases classified as VBIs might have been false positive. The literature is conflicting concerning severity in (partly) vaccinated cases^11^. There are smaller reports of more severe TBE^12^, as well as of comparable clinical severity in VBIs, and of stronger cellular immune responses in VBIs^13^, compared to unvaccinated cases. Larger reports on 54 Austrian and 100 German VBIs reported no evidence of higher severity in VBIs^10,14^. A recent Austrian study including 206 VBIs reported higher severity in VBIs^15^, however the article does not mention diagnostic validation of VBI cases, hence false positive cases might be included in the sample. Further research on diagnostically validated VBIs is necessary.

### Limitations and strengths

Limitations first include that most data were self-reported. We achieved high quality on crucial TBE vaccination variables, as most participants used vaccination cards. Recall bias may, however, have affected retrospectively assessed covariates such as risk behaviour. Secondly, the VE analysis depends on the underlying causal structure. We carefully developed our DAG with expert input to achieve the—to our knowledge—highest validity and report the full DAG and adjustment sets for maximum transparency. Third, only half the vaccinated cases were diagnostically validated. The high false positive rate of 57% among notified vaccinated cases suggests that some of the unvalidated vaccinated cases may have been falsely positive. Such misclassification would have caused a conservative error to VE estimates, hence the true VE might be slightly higher.

Strengths are firstly our uniquely detailed dataset and large sample, allowing for comprehensive VE analysis even in smaller strata of, for instance, heterologous vaccination series. Second, we determined VE with two methodologically different approaches producing overall similar, robust results. The VE estimate determined with the case–control approach is deemed more reliable due to comprehensive confounder adjustment. Third, calculating VE for Germany with a vaccination coverage within range of most European countries suggests results are transferable internationally.

## Conclusion

Our study confirmed very high VE of TBE vaccines at ≥ 3 doses that only decreased slightly when recommended booster intervals were surpassed. VE was lower after only two doses or if the last dose was > 10 years ago. We identified a lack of perceived infection risk and fear of adverse events as main vaccination barriers. Three percent of cases had suspected VBIs; however, most of these were not diagnostically validated. Our results can guide improvement of public health TBE prevention by addressing TBE-specific vaccination barriers and by informing physicians and populations in risk areas about the high effectiveness and safety of TBE vaccination.

## Methods

### Study population and data collection

Routinely notified TBE cases from Bavaria or Baden-Wuerttemberg from 2018 to 2020 meeting the German case definition^34^ were eligible. Local health authorities supported study invitations; cases provided written informed consent. USUMA GmbH recruited German-speaking controls, who had never been diagnosed with TBE and provided verbal informed consent, from a representative telephonic sample. Controls were frequency-matched to cases on age (± 5 years), sex, and 16 geographical regions.

USUMA GmbH conducted standardised 30-min telephone interviews, covering demographics, comorbidities, vaccination barriers, and TBE vaccination (dose number, date and vaccine type for each dose). Participants were asked to have their vaccination card at hand during interviews. If they did not, call-backs for precise vaccination data were offered. Interviews also covered risk factors for TBE infection as well as TBE symptoms and health service utilisation (cases only); for results see Refs.^20,35^. Twenty-three cases were not interviewed; thus, vaccination data originated from hospital discharge summaries and questionnaires completed by cases’ general practitioners. Details on comorbidities and immunosuppression (see Table 3) also derive from these medical sources.

Vaccination data reported in routine surveillance were compared to the information provided in study interviews.

### Definitions of vaccination status

#### Time interval since last dose

This main definition considered dose number and time interval between last dose and symptom onset (cases) or date of data collection (controls). “On-time” vaccination of last dose was defined according to manufacturer’s instructions^30,31^, (Supplementary Fig. 1). As 2 doses are reported to provide protection for up to 1 year^30,31^, 2 doses received 3 weeks to 1 year prior to onset (cases) or data collection (controls) were defined as “2 doses, on-time”. Vaccination dates were partially missing for 6 cases and 72 controls (11.9% of vaccinated participants). Conservative imputation assumed the 28th day of the month; or December 31st where only the year was reported. Sensitivity analysis included only exact dates (Fig. 2).

#### Vaccine type

Homologous vaccination series (≥ 3 doses) with ENCEPUR or FSME-IMMUN was compared with heterologous series, regardless of time intervals. Participants reporting ≥ 1 dose of another vaccine were excluded from vaccine type analysis (*n* = 17, see Supplementary Table 1).

#### Timing of primary immunization

Manufacturer-recommended timing of the first 3 doses (“primary immunisation”) was compared with irregular timing of the first 3 doses, irrespective of vaccine type and time interval. Only participants providing exact dates were used (no imputation). Regular and fast-track immunization^30,31^ were considered with 14-day tolerance margins.

### Data analysis

We report percentages, medians, and means and tested differences with Chi-square tests. P-values < 0.05 were considered statistically significant. Data were analysed in Stata 17^®^.

#### VE based on the case–control approach

Participants not living or spending time in risk areas were excluded (5 cases, 11 controls). Six cases who received their first dose after onset were excluded (see Supplementary Fig. 1). Directed acyclic graphs (DAGs) were constructed in Dagitty^36^ to explore the underlying causal structure and identify minimal adjustment sets for estimating the total causal effect for vaccination on TBE (Supplementary Fig. 2). We calculated adjusted odds ratios with 95% confidence intervals (CI) using multivariable logistic regression. VE was (1 − odds ratio) $\times$ 100%, with corresponding 95% CI. For univariable estimates, see Supplementary Table 1.

#### VE based on the screening method

We applied the formula (as in Ref.^5^):

$$VE \left(\%\right)=100\times \left(1 -\frac{{O}_{v}}{{O}_{u}}\times \frac{{P}_{u}}{{P}_{v}}\right),$$

where O_v_ and O_u_ are observed numbers of vaccinated (v) and unvaccinated (u) cases in the population, and P_v_ and P_u_ are the population proportions of unvaccinated and vaccinated.

P_v_ for ≥ 3 doses on-time, all ages, was derived from routine data for 2019^21^, i.e. 18.98% in the TBE risk areas of the study area. The remaining 81.02% of the population were not differentiated into unvaccinated and incompletely vaccinated, hence based on study data (see Table 1) we assumed that the proportion of incompletely vaccinated persons equalled that of fully vaccinated persons. P_u_ was therefore 81.02–18.98% = 62.04%.

O_v_ and O_u_ were derived from routine TBE surveillance data 2012–2021, when vaccination coverage in the study area was similar^1,37^ to coverage in 2019. Only cases from districts classified as risk areas in 2019 were considered, to ensure the same source population as for P_v_ and P_u_. Fully vaccinated cases were defined as “≥ 3 doses on-time” based on last dose, as described above. A sensitivity analysis assumed 32% misclassification of vaccinated cases as unvaccinated, as observed for cases in routine data (see below).

#### Vaccination barriers

Persons who did not live in or visit risk areas in the past 10 years (5 cases, 11 controls) and cases without interview data (*n* = 23) were excluded from this sub-analysis. To assess barriers, we developed an 11-item tool covering key dimensions of vaccination hesitancy—complacency, confidence, and convenience—based on the 3C model^38^. Participants indicated their degree of (dis)agreement to statements such as “*You were afraid of adverse events*” on a 4-point Likert scale and could name additional reasons.

#### Vaccination breakthrough infections

VBI was defined as TBE infection despite complete (≥ 3 doses), on-time vaccination. We compared TBE manifestations in unvaccinated cases, VBIs, and incompletely vaccinated cases (1–2 doses). Spatial clustering was assessed by mapping cases according to notification district and VBI status (Supplementary Fig. 3). Temporal clustering was visually assessed with histograms of onset dates, split by VBI status.

### Ethics approval

This study was performed in line with the principles of the Declaration of Helsinki. The study was approved by the Ethics Committee of Charité—Universitätsmedizin Berlin, No. EA2/059/18.

### Supplementary Information

Supplementary Information.

## Data Availability

The data presented in this study are available on reasonable request from the corresponding author. The data are not publicly available due to ethical and data privacy protection obligations.
