# Author Correction: Tick-borne encephalitis vaccine effectiveness and barriers to vaccination in Germany

**DOI:** 10.1038/s41598-024-53031-1

**Published:** 2024-01-30

**Authors:** Teresa M. Nygren, Antonia Pilic, Merle M. Böhmer, Christiane Wagner-Wiening, Ole Wichmann, Thomas Harder, Wiebke Hellenbrand

**Affiliations:** 1 Immunisation Unit, Robert Koch Institute, Seestraße 10, 13353 Berlin, Germany; 2 Charité-Universitätsmedizin Berlin, Berlin, Germany; 3 Bavarian Health and Food Safety Authority (LGL), Oberschleißheim, Germany; 4 Institute of Social Medicine and Health Systems Research, Otto-Von-Guericke-University Magdeburg, Magdeburg, Germany; 5 State Health Office Baden-Wuerttemberg (LGA), Stuttgart, Germany

Correction to: *Scientific Reports* 10.1038/s41598-022-15447-5, published online 09 July 2022

The original version of this Article omitted an affiliation for Teresa M. Nygren. The correct affiliations are listed below.

Immunisation Unit, Robert Koch Institute, Seestraße 10, 13353, Berlin, Germany.

Charité-Universitätsmedizin Berlin, Berlin, Germany.

The original article has been corrected.

