# Supplementary material for: Tick-borne encephalitis vaccine effectiveness and barriers to vaccination in Germany
Source: Sci Rep. 2022 Jul 9;12:11706. doi: 10.1038/s41598-022-15447-5 (PMC9271034; doi:10.1038/s41598-022-15447-5)
Supplement: Supplementary file 1 — Supplementary Information. [file 41598_2022_15447_MOESM1_ESM.pdf]

Supplementary Information

Article title: Tick-Borne Encephalitis Vaccine Effectiveness and Barriers to Vaccination in Germany

Journal: *Scientific Reports*

Authors: Teresa M. Nygren, Antonia Pilic, Merle M. Böhmer, Christiane Wagner-Wiening, Ole Wichmann, Thomas Harder, Wiebke Hellenbrand

Corresponding author: Teresa M. Nygren, Robert Koch Institute, Immunization Unit, Berlin, Germany, NygrenT@rki.de

Overview

Supplementary Figure 1. Definition of TBE vaccination status based on time interval since last dose in 575 cases (Panel A) and 975 controls (Panel B)

Supplementary Figure 2. Directed acyclic graph (DAG) of the causal structure underlying TBE vaccine effectiveness

Supplementary Table 1. Univariable estimates for TBE vaccine effectiveness and number of participants with specific outcomes, *n* = 570 cases, 964 controls

Supplementary Figure 3. Spatial distribution of participating TBE cases across the study area (*n* = 581)

Supplementary Table 2. Comparison of characteristics of unvaccinated TBE cases and controls, who lived in or visited TBE risk areas

Supplementary Figure 1. Definition of TBE vaccination status based on time interval since last dose in 575* cases (**a**) and 975 controls (**b**). Manufacturers' instructions foresee booster intervals of 3 years from age 50 for one vaccine and from age 60 for the other. We applied the conservative cut-off at 50 years, irrespective of vaccine type

*6 of 581 cases were previously unvaccinated, but received 1 dose within 30 days after onset (in 5 cases 1–8 days post onset, in 1 case 28 days). These were excluded, hence *n* = 575

a

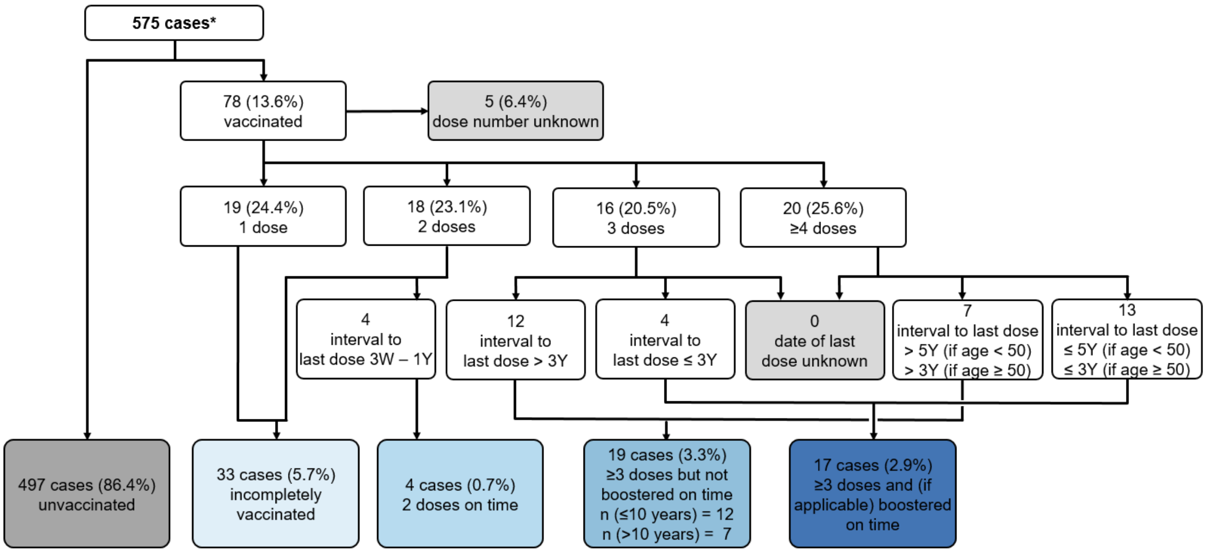

b

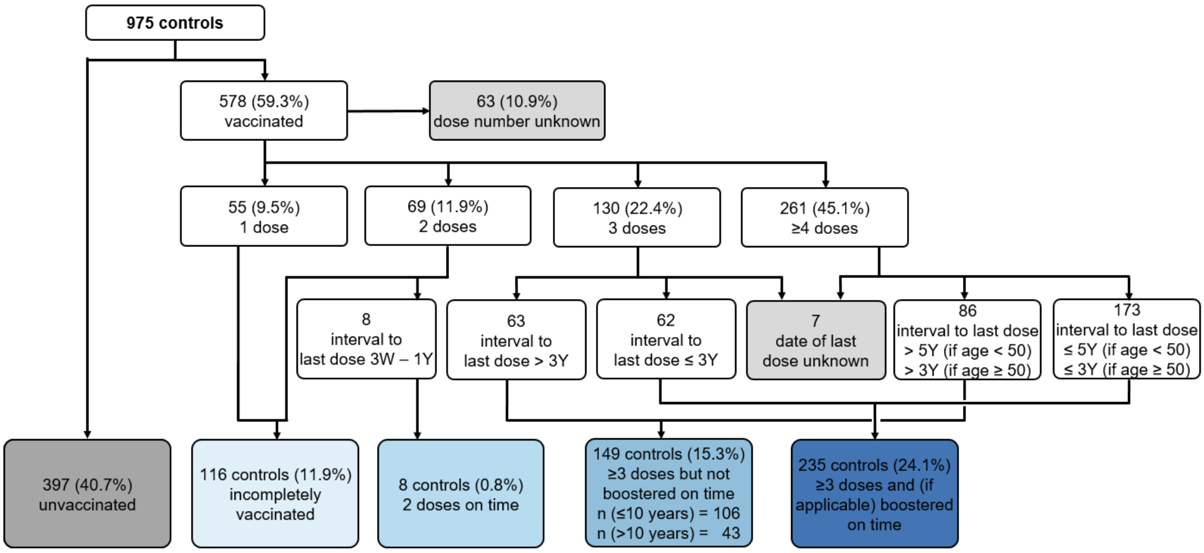

Supplementary Figure 2. Directed acyclic graph (DAG) of the causal structure underlying TBE vaccine effectiveness. Determined by subject-specific knowledge and created with Dagitty [1]

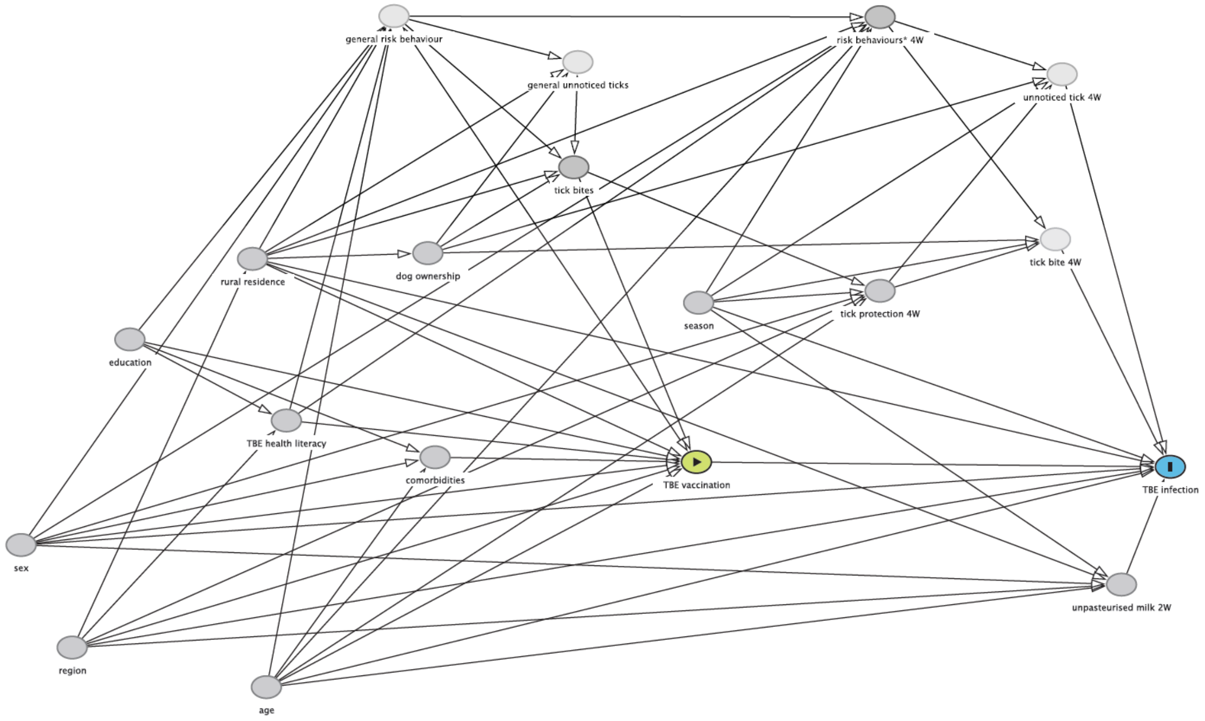

4W = refers to 4-week period of exposure time (cases) or reference period (controls). 2W = refers to 2-week period. Blue node = outcome. Light grey nodes = unobserved parameters. Green node = exposure of interest. The minimal sufficient adjustment set of covariates required to estimate the adjusted total causal effect of TBE vaccination on the outcome included the matching factors (age, sex, region) and the parameters tick bites, dog ownership, risk behaviours 4W (taking walks, gardening, other outdoor activities, not staying on paths), season, and rural residence in settlements with < 5000 inhabitants

* includes taking walks, gardening, other outdoor activities, not staying on paths

Reference

1. Textor J, van der Zander B, Gilthorpe MS, Liskiewicz M, Ellison GT. Robust causal inference using directed acyclic graphs: the R package 'dagitty'. Int. J. Epidemiol. 2016;45(6):1887-94. doi:https://doi.org/10.1093/ije/dyw341

Supplementary Table 1. Univariable estimates for TBE vaccine effectiveness and number of participants with specific outcomes, *n* = 570 cases, 964 controls

**TBE vaccination**		**Number of participants**	**Number of cases**	**VE**	**95% CI lower**	**95% CI upper**
Interval since last dose	Unvaccinated	881	492	1		
	≥ 3 doses, on-time	251	17	94.3%	90.4%	96.6%
	≥ 3 doses, not on-time, ≤ 10 years	118	12	91.0%	83.5%	95.1%
	≥ 3 doses, not on-time, > 10 years	49	7	86.8%	70.3%	94.1%
	2 doses, on-time	12	4	60.5%	-32.3%	88.2%
	1–2 doses	148	33	77.3%	65.8%	84.9%
	≥ 1 dose, additional data missing	75	5	94.4%	85.9%	97.7%
Type of vaccine	Unvaccinated	881	492	1		
	≥ 3 doses ENCEPUR	113	8	94.0%	87.5%	97.1%
	≥ 3 doses FSME-IMMUN	117	13	90.1%	82.1%	94.5%
	≥ 3 doses mixed	108	7	94.5%	88.1%	97.5%
	≥ 3 doses, type unknown	70	8	89.8%	78.4%	95.2%
	< 3 or unknown doses	228	42	82.1%	74.4%	87.5%
	≥ 1 dose TicoVac	17	0	-	-	-
Primary immunisation	Unvaccinated	881	492	1		
	timing as recommended	142	11	93.4%	87.5%	96.5%
	irregular timing	184	12	94.5%	89.9%	97.0%
	exact dates missing	99	13	88.0%	78.3%	93.4%
	incomplete / doses unknown	228	42	82.1%	74.4%	87.5%
Number of doses	Unvaccinated	881	492	1		
	1 dose	74	19	72.7%	53.2%	84.1%
	2 doses	86	18	79.1%	64.2%	87.8%
	3 doses	145	16	90.2%	83.2%	94.3%
	≥ 4 doses	280	20	93.9%	90.2%	96.2%
	≥ 1 dose, additional data missing	68	5	93.7%	84.2%	97.5%
Age group	2–13 years, ≥ 4 doses	14	1	97.1%	75.8%	99.6%
	14–64 years, ≥ 4 doses	201	11	95.4%	91.3%	97.5%
	≥ 65 years, ≥ 4 doses	65	8	86.8%	70.9%	94.0%
Sensitivity analysis: Interval since last dose, no imputed dates	Unvaccinated	881	492	1		
	≥ 3 doses, on-time	211	15	93.9%	89.6%	96.5%
	≥ 3 doses, not on-time, ≤ 10 years	94	10	90.6%	81.6%	95.2%
	≥ 3 doses, not on-time, > 10 years	35	5	86.8%	65.7%	94.9%
	2 doses, on-time	19	5	71.8%	20.9%	89.9%
	1–2 doses	141	32	76.8%	64.8%	84.7%
	≥ 1 dose, additional data missing	153	11	93.9%	88.5%	96.7%

Supplementary Figure 3. Spatial distribution of participating TBE cases across the study area (*n* = 581)

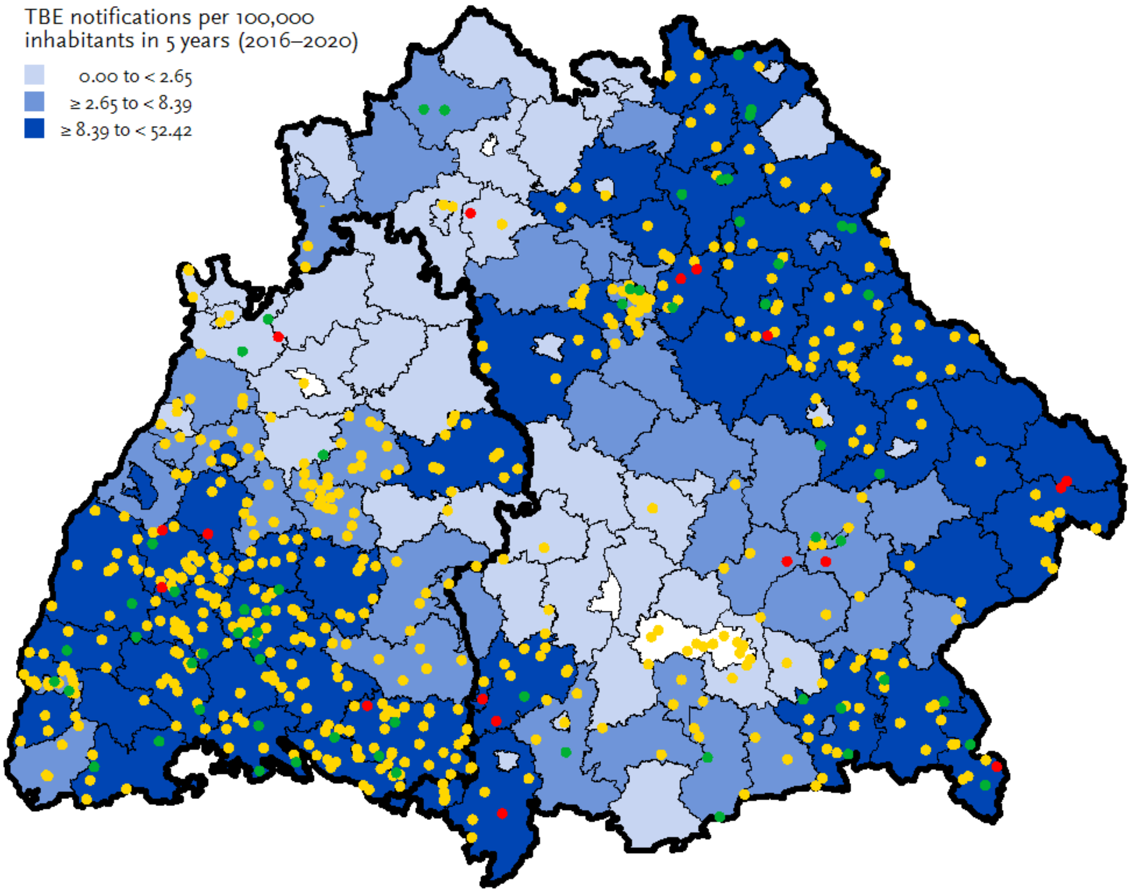

Yellow = unvaccinated cases; Green = cases with partial/expired vaccination or with missing details; Red = cases with vaccine breakthrough infections. Cases were mapped at random points within their district of notification. Background shading in blue reflects TBE incidence 2016–2020 in areas classified as TBE risk areas in 2020. Districts with white shading were not classified as risk areas.

The map was created with RegioGraph Analyse, Version 2018 (GfK GeoMarketing GmbH, https://shop.gfk-geomarketing.de/de/regiograph.html).

Supplmentary Table 2. Comparison of characteristics of unvaccinated TBE cases and controls, who lived in or visited TBE risk areas

	**Cases** *n* = 473 n (%)	**Controls** *n* = 389 n (%)	**p-value**
Age group			
≤18 years	53 (11%)	25 (6%)	
18–64 years	331 (70%)	273 (70%)	0.023
≥65 years	89 (19%)	91 (23%)	
Demographics			
Male	299 (63%)	257 (66%)	0.384
≥1 comorbidity (self-reported)	98 (21%)	96 (25%)	0.166
Home in TBE risk area	462 (98%)	379 (97%)	0.816
Rural residence (< 5,000 inhabitants)	224 (48%)	162 (43%)	0.095
Highest level of completed secondary education* (duration in years)			
Abitur (12–13 years)	138 (29%)	123 (32%)	
Fachabitur (12–13 years)	48 (10%)	21 (5%)	
Realschulabschluss (10 years)	116 (25%)	120 (31%)	0.013
Hauptschulabschluss (9 years)	115 (24%)	94 (24%)	
still in school/none/missing	56 (12%)	31 (8%)	

* English translations: Abitur = general qualification for university entrance; Fachabitur = subject-related entrance qualification; Realschulabschluss = intermediate school-leaving certificate; Hauptschulabschluss = completion of compulsory basic secondary schooling.
